# Optimizing triage and hospitalization in adult general medical emergency patients: the triage project

**DOI:** 10.1186/1471-227X-13-12

**Published:** 2013-07-04

**Authors:** Philipp Schuetz, Pierre Hausfater, Devendra Amin, Sebastian Haubitz, Lukas Fässler, Eva Grolimund, Alexander Kutz, Ursula Schild, Zeljka Caldara, Katharina Regez, Andriy Zhydkov, Timo Kahles, Krassen Nedeltchev, Stefanie von Felten, Sabina De Geest, Antoinette Conca, Petra Schäfer-Keller, Andreas Huber, Mario Bargetzi, Ulrich Buergi, Gabrielle Sauvin, Pasqualina Perrig-Chiello, Barbara Reutlinger, Beat Mueller

**Affiliations:** 1 University Department of Internal Medicine, Kantonsspital Aarau, Tellstrasse, Aarau CH-5001, Switzerland; 2 Emergency Department, Kantonsspital Aarau, Tellstrasse, Aarau CH-5001, Switzerland; 3 Department of Clinical Nursing Science, Kantonsspital Aarau, Tellstrasse, Aarau CH-5001, Switzerland; 4 Department for Neurology, Kantonsspital Aarau, Tellstrasse, Aarau CH-5001, Switzerland; 5 Department of Laboratory Medicine, Kantonsspital Aarau, Tellstrasse, Aarau CH-5001, Switzerland; 6 Clinical Trial Unit (CTU), University Hospital of Basel, Basel, Switzerland; 7 Institute of Nursing Science, University Basel, Basel CH-4056, Switzerland; 8 Department of Psychology, University of Berne, Berne, Switzerland; 9 Department of Hematology, Kantonsspital Aarau, Aarau CH-5001, Switzerland; 10 Morton Plant Hospital, Clearwater, FL, USA; 11 Emergency Department, Hôpital Pitié-Salpétriêre, AP-HP, and UPMC Univ-Paris06

**Keywords:** Triage, Biomarker, Post-acute care needs, Emergency medicine, Manchester triage system

## Abstract

**Background:**

Patients presenting to the emergency department (ED) currently face inacceptable delays in initial treatment, and long, costly hospital stays due to suboptimal initial triage and site-of-care decisions. Accurate ED triage should focus not only on initial treatment priority, but also on prediction of medical risk and nursing needs to improve site-of-care decisions and to simplify early discharge management. Different triage scores have been proposed, such as the Manchester triage system (MTS). Yet, these scores focus only on treatment priority, have suboptimal performance and lack validation in the Swiss health care system. Because the MTS will be introduced into clinical routine at the Kantonsspital Aarau, we propose a large prospective cohort study to optimize initial patient triage. Specifically, the aim of this trial is to derive a three-part triage algorithm to better predict (a) treatment priority; (b) medical risk and thus need for in-hospital treatment; (c) post-acute care needs of patients at the most proximal time point of ED admission.

**Methods/design:**

Prospective, observational, multicenter, multi-national cohort study. We will include all consecutive medical patients seeking ED care into this observational registry. There will be no exclusions except for non-adult and non-medical patients. Vital signs will be recorded and left over blood samples will be stored for later batch analysis of blood markers. Upon ED admission, the post-acute care discharge score (PACD) will be recorded. Attending ED physicians will adjudicate triage priority based on all available results at the time of ED discharge to the medical ward. Patients will be reassessed daily during the hospital course for medical stability and readiness for discharge from the nurses and if involved social workers perspective. To assess outcomes, data from electronic medical records will be used and all patients will be contacted 30 days after hospital admission to assess vital and functional status, re-hospitalization, satisfaction with care and quality of life measures.

We aim to include between 5000 and 7000 patients over one year of recruitment to derive the three-part triage algorithm. The respective main endpoints were defined as (a) initial triage priority (high vs. low priority) adjudicated by the attending ED physician at ED discharge, (b) adverse 30 day outcome (death or intensive care unit admission) within 30 days following ED admission to assess patients risk and thus need for in-hospital treatment and (c) post acute care needs after hospital discharge, defined as transfer of patients to a post-acute care institution, for early recognition and planning of post-acute care needs. Other outcomes are time to first physician contact, time to initiation of adequate medical therapy, time to social worker involvement, length of hospital stay, reasons for discharge delays, patient’s satisfaction with care, overall hospital costs and patients care needs after returning home.

**Discussion:**

Using a reliable initial triage system for estimating initial treatment priority, need for in-hospital treatment and post-acute care needs is an innovative and persuasive approach for a more targeted and efficient management of medical patients in the ED. The proposed interdisciplinary , multi-national project has unprecedented potential to improve initial triage decisions and optimize resource allocation to the sickest patients from admission to discharge. The algorithms derived in this study will be compared in a later randomized controlled trial against a usual care control group in terms of resource use, length of hospital stay, overall costs and patient’s outcomes in terms of mortality, re-hospitalization, quality of life and satisfaction with care.

**Trial registration:**

ClinicalTrials.gov Identifier, NCT01768494

## Background

Hospital emergency departments (ED) are increasingly overwhelmed by patients with both, urgent and non-urgent problems [1,2]. This leads to crowded waiting rooms with long waiting times. As a consequence, patients needing care urgently may not be treated in time, whereas patients with non-urgent problems may unnecessarily receive expensive emergency care. Time to effective treatment is one of the most important predictors for outcomes across different medical conditions (“time is cure”), including patients with septicemia [3], pneumonia [4], stroke (“time is brain”) [5], myocardial infarction (“time is heart”) [6]. For these reasons, a well validated and accurate triage system in the ED is pivotal for an optimal initial triage of medical patients. Moreover, accurate ED triage should not only focus on treatment priority, but also on site-of-care decisions (i.e. outpatient versus inpatient management) and early identification and organization of post-acute care needs.

Different initial triage systems have been proposed including the Manchester triage system (MTS), the Australasian Triage Scale (ATS), the Canadian Triage and Acuity Scale (CTAS) and the Emergency Severity Index (ESI) [7,8]. Among these scores, the MTS is the most widely used score in European and North-American health care settings [7]. The MTS assigns patients to one of 52 flowchart diagrams based on the principal initial presenting complaint. For each of these diagrams red flags are defined based on the clinical presentation and / or vital signs. A triage nurse categorizes patients into different algorithms, and determines treatment priority following a fixed algorithm. Patients are categorized into one of five priority groups (blue, green, yellow, orange, red) with different recommended times for physician assessment (reviewed in Christ et al. [7]).

Only few rigorous clinical studies have investigated the performance of the MTS (and other triage scores) for initial triage decisions. A recent literature review [7] found only four observational studies that have been published today in adult patients with low numbers of included patients (ranging from 50 to 167 patients); although the MTS showed good reliability within these studies, the accuracy of the MTS instrument was suboptimal with only 67% of high risk patients being correctly identified as high priority patients. Thus, there is urgent need for validation in a large, unselected and independent population of medical ED patients and for further refining of the MTS to increase its accuracy. Within the proposed TRIAGE study, we aim to validate the MTS and investigate whether inclusion of vital signs and blood parameters increases its accuracy for both, early identification of high risk patients needing immediate assistance, and patients where delays in initial treatment may not have detrimental consequences.

Initial triage is not only important to assign treatment priorities, but should also assist in estimating the medical risk of patients which influences site-of-care decisions, and post-acute care needs to optimize early planning of post-acute care / nursing support upon hospital admission. This could assist physicians and nurses to make more rational decisions about need for hospital stays and early involvement of social workers to organize the post discharge process (“admission is key to discharge”). For specific diagnoses, such as pneumonia [9], specific medical risk scores have been developed and are propagated by international guidelines to improve initial site-of-care decisions. Yet, there is need for an overall multi-disciplinary risk assessment system to better predict the risk of unselected medical patients and thus need for in hospital management, as well as post-acute care needs at an early stage of ED admission. Obviously, such a comprehensive triage tool can only be developed in close collaboration within the multi-professional team (physician, nurse, social worker).

A promising tool for the Swiss setting was developed in Geneva to predict post-acute institutional care needs and thus assess biopsychosocial risk of patients. As a scoring system at admission and day 3, the post-acute care discharge (PACD) score facilitates discharge planning [10]. A PACD score of ≥8 points on day 3 of hospitalization was accurate to predict discharge to a post-acute care facility (area under the curve [AUC]: 0.82). Data from our institution showed a significant relation between biopsychosocial risk and discharge to a post-acute care facility [11]. The “Selbstpflegeindex” (SPI) is a simple and commonly used nursing and geriatric tool to assess functional dependence in activities of daily life. A SPI score of <32 points indicates a risk for post-acute care deficit [12]. Nurse led care and nurse led units (NLC and NLU) are defined as institutional settings, typically within acute care hospitals, which provide independent specialized nursing service for post-acute care patients, who need predominantly nursing care. They constitute a possible model of care for patients with low medical yet high nursing risk [13,14] and are characterized and operationalized by five factors: 1) inpatient environment offering active treatment; 2) case mix based on care needs; 3) nursing leadership of the (multidisciplinary) clinical team; 4) nursing conceptualized as the predominant active therapy; 5) nurses’ authority to admit and discharge patients [13,14]. There are indications that post-acute care patients discharged from NLUs have a better functional status and greater psychological well-being, are more often discharged home than to another institution and less often readmitted to the hospital than patients receiving usual care. There are also indications that these patients are more satisfied with care [14-16]. Within the proposed TRIAGE study we aim to validate and further improve these nursing / care scores to enable more wide-spread adoption for optimized patient management.

Discharge planning has to begin on admission. We and others have previously investigated the utility of different blood biomarkers for an optimized prognostic assessment in patients presenting to the ED with respiratory infections [17-26], sepsis [17,27], acute heart failure [28-30] and myocardial infarction and other important medical conditions. Among different markers, pro-adrenomedullin (proADM) has generated interest as an accurate prognostic marker for adverse outcome with high validity across different medical situations [17,18,27-30]. We also investigated biopsychosocial factors, which influence admission and discharge decision and are thus prerequisites for clinically meaningful site-of-care decision making [31,32]. Reducing the number of in-hospital days is important not only for cost issues. Hospital-acquired disability is an emerging issue in health care and older, frail medical patients at high risk for allegedly premature referral to a nursing home with consecutive depression and further deterioration of mental and physical independence [33]. To improve hospital management of patients with lower respiratory tract infections, we have developed a biomarker-enhanced clinical risk score (combining the CURB65 score and proADM) [34,35]. The efficacy and safety of this score was recently tested in a randomized controlled trial at the Kantonsspital Aarau. Based on these studies focusing on respiratory infections, we hypothesize that adding clinical parameters and prognostic biomarkers to an established triage risk score, such as the MTS, at the very proximal time point of ED admission, has a substantial and clinically relevant potential to improve its performance and translate into better triage of patients on admission and during hospitalization. This will help to identify both, high risk patients in need of urgent care and inhospital management and low risk patients where longer waiting times have no detrimental consequences and who can potentially be treated in outpatient, NLC, post-acute or nursing home settings.

Importantly, previous efforts to validate and improve current triage scores in unselected patients across different medical diagnoses presenting to the ED were limited by the isolated focus on the ED, a small sample size and / or small spectrum of medical conditions, and observational “hypothesis-generating” designs only. In addition, no study has investigated whether initial measurement of blood biomarkers and/or clinical parameters has the potential to improve patient triage. Thus, a large-scale comprehensive study is warranted to validate previous findings, investigate whether prognostic markers and clinical parameters could improve patient triage from admission to discharge and translate these findings into a new, improved initial triage system for use in routine clinical care throughout the hospital stay. Importantly, we aim to not only focus on medical risk, but also include biopsychological risk scores for post-acute care / nursing needs to enable a more comprehensive assessment of a patient’s situation.

Such an enhanced initial patient assessment that supports a clinician’s ability to accurately triage and risk stratify patients has the potential to facilitate early and appropriate therapeutic interventions and prevent unnecessary waiting times, improve important initial triage decisions in regard to site-of-care decisions, help recognize and plan post-acute care needs early for immediate social worker involvement, reduce duration of hospital stays and, overall, optimize allocation of health-care resources, and at the same time decrease mortality and morbidity by focusing the medical attention to high risk subjects. As part of an ongoing prospective and large-scale research effort, we plan to later evaluate the efficacy and safety of this new triage algorithm in a second cluster-randomized controlled trial (comparing the new algorithm with an usual care control group).

## Methods/design

### Overall hypothesis and research aim

The overall hypothesis of this study is that an improved initial triage of patients at an early stage of ED admission with incorporation of the MTS, initial clinical parameters and vital signs, prognostic blood markers and the PACD score [10] will improve patient triage and translate into more objective estimation of triage priority, need for hospitalization and post-acute care needs. In this initial study we aim to derive a three-part triage algorithm, which will subsequently be evaluated in a second randomized controlled trial.

### Specific aims

To derive a three-part triage algorithm to better predict (a) treatment priority; (b) medical risk and thus need for in-hospital treatment; (c) post-acute care needs of patients at the earliest time point of ED admission in a large and unselected population of medical patients.

**Figure 1:**
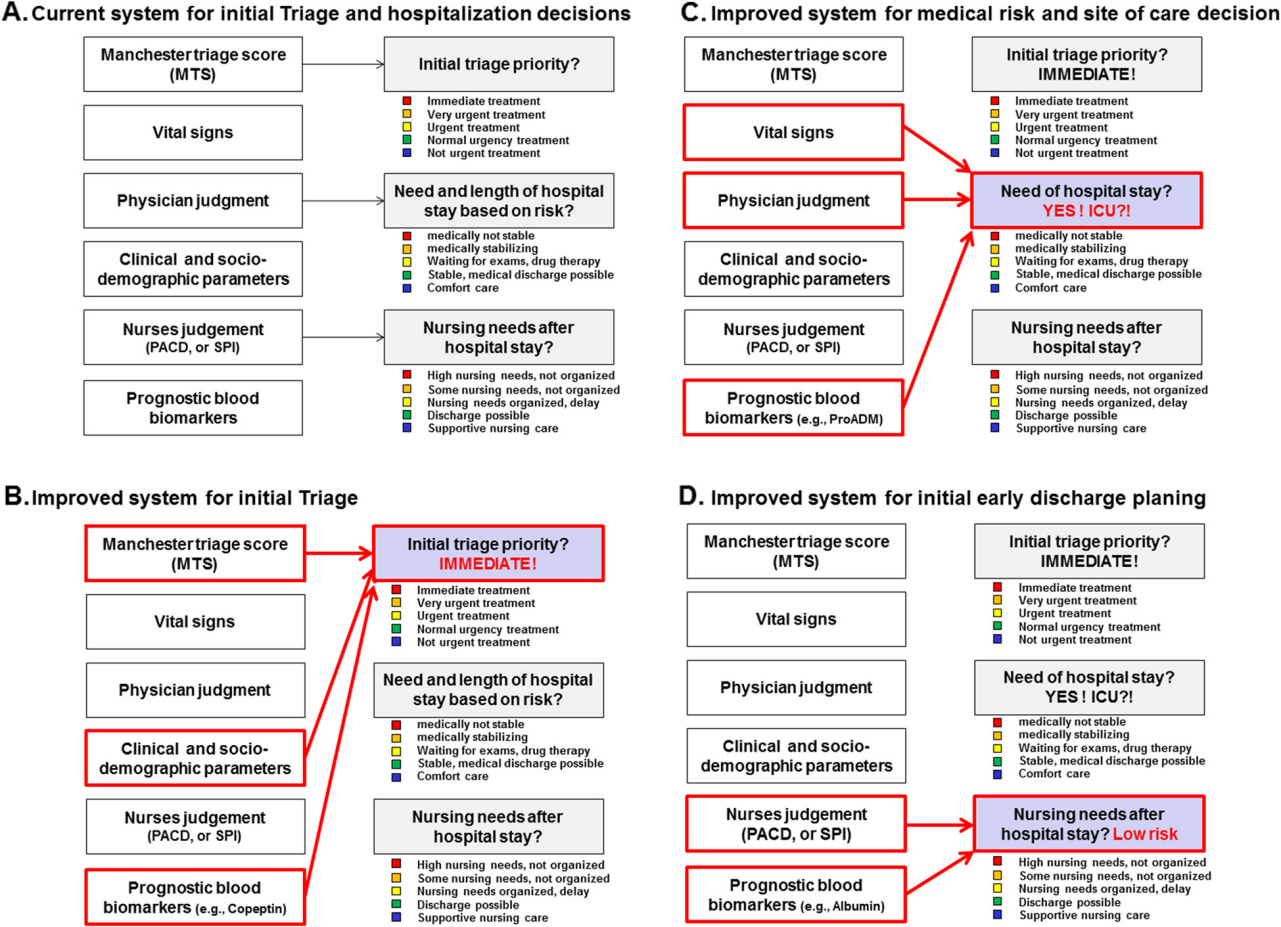
**Patient assessment for improved triaging of initial triage priority (Figure B), need for in hospital treatment (Figure C) and care needs (Figure D).** Figure **A** shows the current conventional approach**.**

This is done by development of three algorithms for assessing:

(a) Treatment priority (high vs. low priority). This will be based on the MTS as the current state of the art tool, and other clinical variables and blood biomarkers (Figure 1B). This algorithm should help to correctly prioritize patients in a crowded ED setting and allocate resources to patients needing them first.

(b) The overall 30 days medical risk based on different initial socio-demographic parameters, initial complaints, clinical parameters, vital signs and blood biomarkers across different medical conditions. This will help physicians to objectively estimate the need for inpatient treatment in patients and may improve site-of-care decisions (Figure 1C).

(c) The risk for post-acute care needs, i.e. if patients need to be transferred to post-acute care institutions. This may improve early discharge planning (Figure 1D).

### Study design

This is a prospective, observational, multi-center, multi-national cohort study. Over the time course of 12 months, we will prospectively include all consecutive medical patients seeking ED care. As an observational quality control study, the Institutional review board (IRB) of the Canton of Aargau has approved the study and waived the need for informed consent (EK 2012/059).

### Setting, patient population, inclusion and exclusion criteria

We will conduct this study in an multi-center, multi-national inter-professional and interdisciplinary collaboration at the Kantonsspital Aarau (Switzerland) including the Medical University Department, the Emergency Department, the Center of Laboratory Medicine, and the Clinical Nursing Science Department, as well as the Clinical Trial Unit (CTU) of the University Hospital of Basel and the Institute of Nursing science of the University of Basel; as well as the Emergency Department, Hôpital Pitié-Salpétriêre in Paris (France) and the Morton Plant Hospital in Clearwater, Florida (USA). Depending on the availability, we will also include other clinical centers to validate our findings.

We will include all consecutive medical patients including patients with neurological admission diagnoses presenting to ED for medical reasons and follow them during the hospital course until hospital discharge. There will be no exclusions except for non-adult and non-medical patients.

### Clinical information and assessment outcomes

We will record initial vital signs (i.e. blood pressure, respiratory rate and others) and clinical parameters (i.e. main complaint, initial diagnosis) in the ED and collect left over blood samples in all patients. Clinical information including socio-demographics and comorbidities, patient outcomes and nursing information using the “Selbstpflegeindex” (SPI) and the PACD will be assessed prospectively until hospital discharge using the routinely gathered information from the hospital electronic medical system used for coding of Diagnosis-Related Groups (DRG) codes. This already available information supports the reliable assessment of baseline characteristics including demographics, comorbidities, acute medical conditions requiring the ED visit and different patient outcomes including inhospital mortality, resource use in terms of admission to the intensive care unit, length of stay (LOS) in the hospital and overall costs. We will also collect information about care needs in case of transfer to another post-acute institution after hospital discharge.

We will contact all patients by phone interview 30 days after admission to evaluate vital and functional status, care needs at home, rehospitalisation rates, satisfaction with care, preparedness for discharge,quality of life measures using the EQ-5D questionnaire [36] and EQ VAS among others.

### Daily assessment of clinical stability with the “Visitentool”

We will assess clinical stability of patients daily during the medical rounds. We have developed an online computer-based stability assessment tool - called “Visitentool” – where patient’s stability and readiness for hospital discharge must be entered daily on clinical rounds. Similarly to the MTS, this is done in five categories (medical red: not stable, orange: stabilizing, yellow: stable but elective procedure awaiting, green: stable, discharge possible, blue: terminal/palliation) (nursing red: biopsychosocial risk (PACD ≥8) and/or post-acute care need likely, orange: interventions planned, yellow: ready for discharge/transfer but delay, green: discharge/transfer possible, blue: terminal) (social red: social services involved/in process, orange: external placement done, yellow: definitive date set for external relocation with time lag, green: definite date for external relocation set, date corresponds to earliest possible date regarding clinical stability). Importantly, physicians, nurses and social workers assess clinical stability and readiness for discharge daily from their perspective with this online tool to better understand the time to medical stability and readiness for discharge, and to study delays in hospital discharge which will also be documented.

### Endpoints

**Figure 2:**
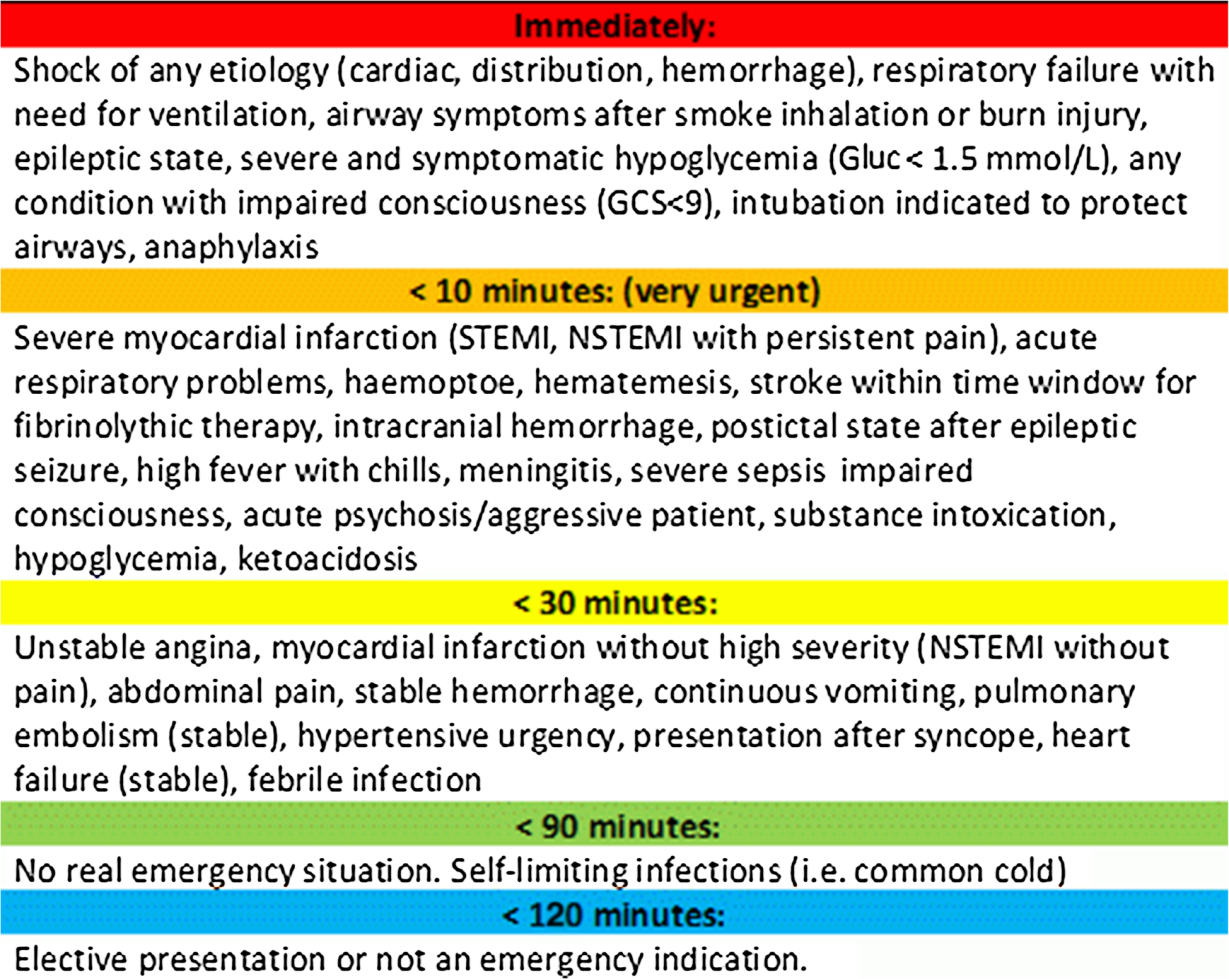
**Guidelines for adjudication of initial treatment priority with practical examples.** The main question for adjudicators will be “*under difficult circumstances, what is the maximum possible time that this patient would have been able to wait before being seen?*” adapted from on a previous study [37].

To improve management of patients at the earliest time point of ED admission, we aim to develop a triage algorithm based on three distinct decision rules for (a) assessment of triage priority, (b) need for hospitalization and (c) post-acute care needs as shown in Figure 1. We therefore have three distinct main endpoints:

(a) Initial triage priority adjudicated by two independent ED physicians. Similar to a previous study [37], the physicians will evaluate what the real degree of urgency (“Goldstandard”) would have been, based on the ED data, results of diagnostic tests, and the final diagnosis. Specifically, the main question for the adjudicators will be “*under difficult circumstances*, *what is the maximum possible time that this patient would have been able to wait before being seen*?” with options of “patient could not wait”, 10 minutes, 30 minutes, 90 minutes, or 3 hours. To further standardize the adjudication, we have developed examples as demonstrated in Figure 2. We will collapse the initial 5 priority categories into 2 categories (i.e. low [more than 10 min, class 3, 4 or 5] vs. high priority [less than 10 min, class 1 or 2]). The 2 adjudicators will answer this question in regard to a medical prognostic focus and to a patient comfort focus (i.e. pain). In case of disagreement, a third independent physician will review the case until consensus is reached.

(b) Adverse 30 day outcome (death, intensive care unit admission or unplanned hospital re-admissions) within 30 days following ED admission.

(c) Post-acute care needs immediately after hospital discharge. This will be defined as transfer of patients to a post-acute care institution (i.e. transition to a nursing home, rehabilitation center and others).

Other endpoints will be defined as follows

• Time to first physician contact as assessed in the nursing chart; we will investigate this endpoint stratified by patients’ risk, i.e. we will compare time to first physician contact in high-triage-priority and low-triage-priority patients and stratified by different diagnoses.

• Time to initiation of adequate medical therapy in predefined subgroups (e.g., antibiotic therapy for infections, door to needle time for myocardial infarction; early goal directed therapy in sepsis patients, pain relief medication in patients presenting with pain, blood pressure control in patients with a hypertensive crisis); we will further assess time to discharge from the ED to the ward.

• Satisfaction with care, preparedness for discharge, need of care at home, functional status and quality of life as assessed in the day 30 telephone interview.

• Overall hospital costs as assessed by the electronic medical records.

### Procedures and management of patients throughout the trial

All patient procedures are part of routine clinical care. Upon ED admission, a triage nurse will assess triage priority according to the MTS. Vital signs will be recorded and left over blood samples will be stored for later batch analysis of blood markers. The risk for post-acute care needs will be assessed with the PACD score per usual care. Patients will be reassessed daily during the hospital course for medical stability and readiness for discharge with an electronic tool as defined above (Visitentool). To assess patient outcomes, data from electronic medical records and from a patient quality questionnaire complemented with follow-up interviews at day 30 will be used. Below the detailed different steps of patient management are shown.

Step 1. Upon ED admission, all patients will be assessed by a designated triage nurse. MTS triage priority will be assigned based on the MTS as recommended [7]. This will be entered into the clinical information system along with information about main complain, vital signs and clinical variables. The triage nurse will also assess the PACD on admission.

Step 2. In all patients, the triage nurse will perform a standardized blood draw for routine measurement of blood chemistry per usual care; left over samples will be aliquoted at the center of laboratory medicine and used for later batch analysis of biomarkers.

Step 3. Upon ED discharge, the attending ED physician will adjudicate a medical triage priority based on all medical results available at this time to all patients (high vs. low triage priority).

Step 4. Throughout the hospital stay, patients will be managed by physicians, nurses and social care in accordance to hospital guidelines according to the underlying medical condition. This will be at the discretion of the treating physicians, nursing and social worker staff, independent of the research team. During hospitalization, nursing scores will be collected per usual care and entered into the electronic medical system along with information about the planed care provided to patients after hospital discharge.

Step 5. All patients will be contacted 30 days after hospital admission for a telephone interview with a predefined questionnaire to assess vital and functional status, hospital readmission, as well as quality of life, care needs at home and satisfaction with care provided.

### Blood draws and candidate biomarkers

Left over blood samples of routinely collect blood tubes on admission will be immediatly centrifuged, aliquoted and frozen at -20C for later batch analysis of blood various biomarkers. The results of this analysis will not be available at the time of hospitalization of the patients and, thus, physicians and patients will be blinded to their results.

We will examine blood markers from different distinct biologic pathways as candidate biomarkers. Thus, we will assess markers of infection, inflammation, organ dysfunction, endothelial dysfunction, vasodilation / infection-control, stress hormones, cardiac dysfunction, nutrition, and kidney function, which all have been shown to predict adverse outcomes in different types of medical conditions (Table 1). Depending on the expected benefit from a literature research, the available funding and logistic support, we will decide which markers should be analyzed in the stored aliquots.

**Table 1:** Candidate parameters for improved diagnostic and prognostic patient assessment

**Candidate parameters**	**Pathophysiological concept / Previous research findings**	**Ref.**
**Infection marker** (PCT)	CALC I-gene associated hormokine of bacterial infections; correlates with infection severity and risk for bacteremia; responsive over time; established for antibiotic stewardship in respiratory tract infections and sepsis; moderate prognostic accuracy	[38-46]
**Inflammatory markers** (CRP, WBC)	Increase in response to inflammation and infection; low specificity and moderate sensitivity; low prognostic accuracy	[47-49]
**Organ dysfunction markers** (Lactate, coagulation, liver)	For progression of sepsis to severe sepsis with organ dysfunction; lactate is the recommended biomarker for early goal directed resuscitation therapy	[50-52]
**Endothelial activation markers** (VCAM-1, ICAM-1, E-selectin, PAI-1, sFLT-1, ET-1)	Marker panel correlates with vascular dysfunction, with sepsis severity and sepsis-related mortality; highest markers in septic shock; marker are dynamic over time and drop when patients condition is improving	[51,53-58]
**Vasodilation / infection markers** (Pro-adrenomedullin)	CALC V-Gene associated hormokine with high prognostic accuracy in pneumonia and sepsis in the ICU setting; significantly improves pneumonia risk scores (PSI, CURB65) based on OPTIMA II study	[17,18]
**Stress markers** (vasopressin precursor [copeptin], cortisol)	High prognostic accuracy in respiratory infections and sepsis; significantly improve previous pneumonia risk scores (PSI, CURB65)	[18,59,60]
**Cardiac dysfunction markers** (Natriuretic peptides: BNP, )	Correlate with cardiac dysfunction / cardiovascular stress; moderate to high prognostic accuracy	[61,62]
**Kidney dysfunction** (Urea, creatinine, NGAL)	High correlation with kidney dysfunction and increase in (pre) shock; also correlate with (septic) kidney injury	[63]
**Blood cells** (red cell distribution width)	Measure of variability of red cells; associated with in-hospital and ICU mortality	[64-66]
**Nutrition** (Albumin, pre- albumin, vitamin D)	Markers of nutrition have been shown to correlate with the general condition of patients and the risk of needing nursing care.	[67]

### Ancillary projects

Within this study, we have several ancillary projects focusing on different aspects of patient care in this medical population.

First, we will look at costs from different perspectives, i.e. patient, society perspective, insurance perspective and hospital perspective. We will collect detailed cost data as well as resource use data. Based on the daily clinical assessment we will have good estimates how length of stay (LOS) could be reduced in patients without increasing their risk, i.e. at the time patients are classified as “medically stable” by the treating physician team. We will develop cost models using DRG reimbursements to evaluate the potential in savings.

Second, within a subset of patients we will focus on psychological distress defined as negative psychological reaction which may pre-exist or develop in the context of an acute disease potentially involving a variety of affective, cognitive, and behavioral reactions, such as fear, sadness, anxiety, frustration, or non-compliance. In this ancillary project we aim to explore the prevalence and course of patients’ psychological distress on ED admission and within the hospital stay. To measure psychological distress we will use several validated instruments including the Distress Thermometer (DT) [68,69] and the positive and negative affect schedule (PANAS) [70]. Beside general distress our focus will particularly lie on anxiety and depression assessed with the Patient Health Questionnaire-4 (PHQ-4) [71]. Additionally we will explore the relation of psychological distress with health outcomes (mortality, comorbidity, health-related quality of life, LOS among other) 30 days after admission. Finally, we aim to further delineate the role of specific patient’s psycho-social resources (personality, social support, age, sex, SES, medical diagnosis) with regard to distress and health outcomes.

### Statistical considerations and sample size

The purpose of this study is to develop an improved triage tool based on three distinct algorithms for (a) estimation of treatment priority (model 1), (b) prediction of medical risk (model 2) and (c) risk of post-acute care needs (model 3). For this purpose we have defined three distinct binary endpoints (i.e. high vs. low triage priority, adverse medical outcome within 30 days, post-acute care need) for which independent prediction rules will be developed using a similar approach for each one. However, based on the published literature, different candidate parameters will be considered as predictors for inclusion into the models.

In brief, for each algorithm we will select a parsimonious set of parameters from a comprehensive list of candidates including vital signs, clinical / socio-demographic predictors, blood markers, the MTS and the PACD. For blood markers we will focus on proADM and urea as the most established prognostic markers; however, we will also consider other markers for completion based on the availability of routine data (Table 1). We will use multivariable logistic regression analysis and different selection techniques including stepwise regression, Lasso among others [72]. We will also compare the non-parametric CART analysis to decide if a simpler algorithm would qualify. Improvements in the area under the receiver operating curve (AUC) and reclassification statistics will inform about the benefit of adding parameters to the model [72,73]. We will apply split sample validation (training and validation set with a ratio of 1:1) and present goodness of fit statistics to assess robustness and internal validity. Based on these results, we will derive weighted admission risk scores for the three main models, which can be used for later decision making (Figure 1). We will also look at subgroups to investigate differences in performance between main diagnoses and socio-demographic factors (age, gender) by inclusion of interaction terms into the logistic models.

For our model 1 (treatment priority), we will use adjudicated initial triage priority as the endpoint of interest (low vs. high triage priority) as defined above. As the MTS is well established for this purpose, we will first investigate the ability of the MTS to identify high priority subjects. We will then investigate whether addition of clinical parameters, vital signs and blood markers improve the MTS using statistical approaches outlined above. In a second step, we will investigate the performance of the MTS in subgroups of patients, i.e. stratified by initial admission diagnosis (e.g. myocardial infarction, congestive heart failure, infection, falls, lung embolism), by main clinical complain (e.g. dyspnea, fever, cough, pain) and by age quartiles, we will include interaction terms to study whether the association of the MTS and / or biomarkers varies across subgroups (effect modification). If significant effect modification is found, we will adapt the risk score to certain admission diagnoses.

For our model 2 (adverse outcome within 30 days) we will focus on death or ICU admission as the main outcome, in accordance with established risk scores (such as the pneumonia severity index or the CURB65 score) [9]. In previous research [18,27,34,35] we found that specific blood biomarkers (i.e. proADM) have very high prognostic accuracy in the range of clinical risk scores, and that this is true across different medical conditions. However, other “baseline” factors, such as age and comorbidities are likely providing prognostic information beyond that of blood markers. Thus, it is a promising approach to combine these factors in a combined risk model.

Our model 3 (post-acute care needs) will focus on care needs in patients after hospital discharge. The PACD score was developed for this purpose. However, the PACD focuses mainly on care needs of patients prior to hospital admission and availability of help in the home setting, but not as much on the current medical situation. It is therefore possible that addition of parameters reflecting the severity of disease (vital signs, blood markers) or the nutritional condition (blood markers) further improves its accuracy. We will therefore start with the PACD and investigate whether addition of other parameters significantly improves its accuracy as outlined above.

We aim to include a total of at least 5000 patients over the course of 12 months, with expected rates for high treatment priority of 20% (n=1000), for adverse outcomes of 10% (n=500) and for post-acute care needs of 20% (n=1000). This will provide 50–100 degrees of freedom for each model (with 10 cases in the data set per degree of freedom in the statistical model), and thus high power for the calculation of the main multivariate models overall, in pre-defined subgroups and after inclusion of interaction terms.

## Discussion

### Potential limitations and bias

Treatment priority as adjudicated by the attending physicians at ED discharge is not a “hard” endpoint and may be subject to variation due to different levels of experience of physicians. Nevertheless, we have developed guidelines (Figure 2) that will help to standardize adjudication based on previous research in this field [37]. In addition, we will also look at other more objective endpoints (i.e. mortality, ICU admission, LOS). We will also collect information about physicians (years of experience, age, baseline “opinion” about risk scoring) and will thus be able to adjust the analysis accordingly. Also, physicians and nurses will not be blinded to the MTS, PACD and the risk assessment overall and thus may adapt their priority recommendation accordingly. This may overestimate the performance of the triage scoring systems. In terms of other blood markers and clinical parameters to improve the MTS, this bias will be minimal. Within this observational quality control project, we will not be able to demonstrate whether improved triage of patients translates into better management and improved outcomes; for this reason, we plan a second randomized controlled trial. While most prognostic blood markers (including proADM) are now commercially available within 1–3 hours, faster point-of-care tests are currently being developed that would enable measurement of marker within minutes, similar to a glucose measurement. This will further improve bedside use of these markers in the near future trial.

### Significance and outlook

Patients presenting to the ED currently suffer from delays in initial treatment due to suboptimal triage. Using a reliable initial triage system is an innovative and persuasive new approach for a more targeted management of patients in the ED. The proposed TRIAGE study has realistic and substantial potential to improve triage and thereby management of patients from admission on the ED throughout their hospital stays. We hypothesize that accurate prediction of medical risk and early recognition of care needs (i.e. using the PACD and scores) may facilitate early discharge planning, and thereby reduce hospital-acquired disability [33] and LOS.

In light of the current discussion about our limited health care resources, the proposed TRIAGE study has high relevance for the Swiss, French and US helath care systems health care system. As hospital stays are very costly, any shortening will yield large savings (≥ CHF 1000 per day and patient). Just in time after the introduction of the “Swiss DRG” [74], our analysis will bring valuable insight into imminent challenges for the healthcare system, also in terms of cost and the rational allocation of our limited health care resources. Most importantly, risk-appropriate triage is expected to free urgently needed capacity for acutely-ill medical patients.

Based on the results of this study, we will propose a randomized controlled trial to test the efficacy and safety of the herein derived optimized triage algorithms.

### Trial status

Ongoing trial with start of recruitment in June 2013 and planned termination 12 month later.

## Abbreviations

CI: Confidence interval; ED: Emergency department; ICU: Intensive care unit; LOS: Length of stay; MTS: Manchester triage system; OR: Odds ratio; PCT: Procalcitonin; ProADM: Pro-adrenomedullin; DRG: Diagnosis-related groups; SPI: Selbstpflegeindex (self care index); PACD: Post-acute care discharge score.

## Competing interests

This study is supported in part by the Gottfried and Julia Bangerter-Rhyner-Foundation, the Swiss Academy for Medical Sciences (SchweizerischeAkadmie der MedizinischenWissenschaften [SAMW]), the Medical University Department of the KantonsspitalAarau, and Thermo Fisher Scientific. DrsSchuetz, Hausfater, Amin and Mueller received support from Thermo Fisher Scientific. All other authors declare that they have no competing interests.

## Authors’ contributions

PS, PH, DA, SH, LF, SDG, AC, PSK, BR and BM had the idea for the study and designed the study protocol. All authors amended and commented on the manuscript revising it critically for important intellectual content. All authors read and approved the final manuscript.

## Pre-publication history

The pre-publication history for this paper can be accessed here:

http://www.biomedcentral.com/1471-227X/13/12/prepub
